# Insights into scleral violaceous hue in anterior scleritis: anterior segment optical coherence tomography evaluation

**DOI:** 10.1007/s00417-025-06788-8

**Published:** 2025-03-17

**Authors:** Plern Sutra, Thananop Pothikamjorn, Sarah Lopez, Jaskirat Takhar, Mathinee Chongchareon, Jeremy Keenan, John A. Gonzales

**Affiliations:** 1 Francis I. Proctor Foundation, University of California, San Francisco, USA; 2 Department of Ophthalmology, Vajira Hospital, Navamindradhiraj University, Bangkok, Thailand; 3 Faculty of Medicine, Chulalongkorn University and King Chulalongkorn Memorial Hospital, Thai Red Cross Society, Bangkok, Thailand; 4 Department of Ophthalmology, University of California, San Francisco, CA 94158 USA; 5 University of Hawaii, John Burns School of Medicine, Honolulu, HI USA; 6 Department of Ophthalmology, Case Western Reserve, Cleveland, OH USA; 7 Bangkok Metropolitan Administration General Hospital, Bangkok, Thailand

**Keywords:** Anterior segment OCT, Scleritis, Anterior scleritis, Violaceous hue, Violaceous sclera, Scleral thickness

## Abstract

**Purpose:**

To determine the scleral thickness of inactive scleritis characterized by a violaceous hue (violaceous sclera) using anterior segment optical coherence tomography (AS-OCT).

**Methods:**

Retrospective observational case series of patients with inactive unilateral anterior scleritis featuring a violaceous hue. Mean scleral thickness was measured by AS-OCT in violaceous areas and compared with the same region in the contralateral unaffected eye. Measurements were performed by two masked graders.

**Results:**

Nine patients with median age of 52 ± 12.8 years were assessed. Eight patients were female. Rheumatoid arthritis and history of treated latent tuberculosis (33.3%) were the most common causes of anterior scleritis. Mean scleral thickness was 582.93 ± 124.03 µm and 648.59 ± 103.61 µm for violaceous sclera and the corresponding unaffected areas of the contralateral eye, respectively (mean difference = -65.65 µm, 95% CI: -143.73 to 12.42, *p* = 0.0885). The mean image contrast percentage of scleral hyperreflectivity as assessed by image conversion in an area of violaceous hue was 65.07 µm ± 6.49 µm compared to 42.70 µm ± 5.46 µm of unaffected areas (mean difference = 22.37 µm, 95% CI: 14.72 µm to 30.03 µm, *p* = 0.0001).

**Conclusion:**

Using AS-OCT, the thicknesses of violaceous sclerae were not significantly thinner than the contralateral unaffected areas, despite a mean difference of approximately 65 microns. The increased scleral hyperreflectivity observed in the violaceous sclera may suggest collagen remodeling in these areas. Such remodeling could play a role in the sclera reflecting violaceous hues while still preventing direct visualization of the underlying choroid.

## Abstract (highlights)

**Key messages:**

*****What is known***:**

- The violaceous sclera observed in scleritis is believed to result from an inflammatory process leading to scleral thinning, potentially exposing the underlying choroid.

*****What is new***:**

- Our study reported the thickness of the violaceous sclera was not significantly different from that of the contralateral unaffected areas.
- We found that the increased scleral hyperreflectivity observed in the violaceous sclera may suggest collagen remodeling in these regions.

## Introduction

The development of the characteristic violaceous hue following the quiescence of active anterior scleritis has been suggested to be due to the ability to see the underlying choroid [1–4]. Others have suggested that post-inflammatory remodeling of the sclera results in the reflection of blue light [5]. Indeed, electron microscopy shows that normal sclera features an organized arrangement of collagen and proteoglycans, which can become disrupted in scleritis [6, 7].

We wished to investigate whether areas of inactive scleritis characterized by a violaceous hue were thinner compared to the contralateral unaffected area by using enhanced depth imaging (EDI) scleral mode in anterior segment optical coherence tomography (AS-OCT). Additionally, we sought to determine whether there was greater hyperreflectivity in areas of violaceous hue compared to the contralateral unaffected area, which could suggest remodeling of the sclera in the affected regions. We hypothesized that there would not be a significant difference in scleral thickness between the two areas of sclera and that AS-OCT would demonstrate that the remaining sclera in the area of violaceous hue would still be thick enough to prevent visualization of the underlying choroid.

## Materials and methods

A retrospective study was performed identifying patients with inactive unilateral anterior scleritis seen at the Francis I. Proctor Foundation between January 1, 2017, to December 31, 2018. Progress on the project was delayed due to the COVID-19 pandemic in 2019, with data analysis resuming only recently. Potential cases were identified from a chart review using International Classification of Diseases (ICD9/10) codes for anterior scleritis. We had developed a protocol for obtaining anterior segment OCT images of areas in inactive scleritis characterized by a clinical feature of violaceous hue and comparing that region to the same scleral area in the contralateral eye. The study was approved by the Human Research and Ethics Committee at the University of California, San Francisco and adhered to the tenets of the Declaration of Helsinki.

The inclusion criteria were defined by (1) the presence of violaceous area in a patient with inactive unilateral nodular or diffuse scleritis located anterior to the equator and (2) with no active ocular disease in the unaffected contralateral eye (unilateral scleritis). Inactive scleritis was confirmed clinically by a trained uveitis specialist (JAG). Patients with an AS-OCT scan demonstrating active scleral inflammation (i.e., hyporeflective pockets/spaces that represent intrascleral edema) were excluded [8, 9]. Patients were also excluded if they were under the age of 18 years, had necrotizing scleritis, had previous injury or surgery involving the sclera, or had high myopia (more than −6.00 D) [10].

## Imaging and imaging analysis

**Fig. 1:**
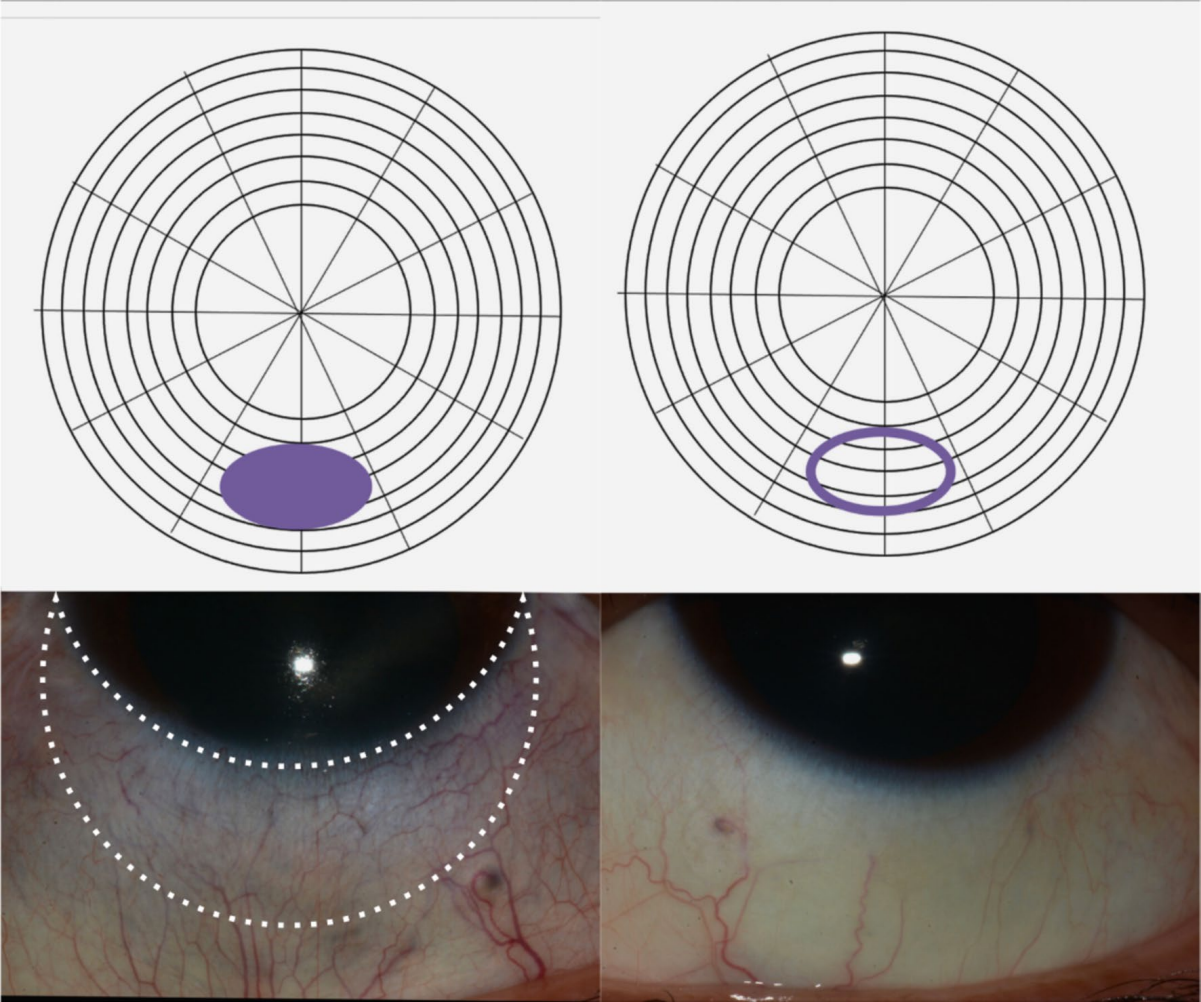
The upper panel demonstrates the clinical image of the affected scleral region alongside the corresponding contralateral unaffected area. The lower panel provides a schematic diagram of the sclera, with the innermost circle representing the cornea and the outer circle representing the sclera. Each concentric space corresponds to 1 mm of distance. The dense circle marks the affected scleral region, while the donut-shaped circle denotes the contralateral unaffected sclera

All 4 quadrants of the globe (nasal, temporal, superior, inferior) in each eye were photographed using external photography (Canon EOS DSLR camera). Patients with scleritis at the Francis I. Proctor Foundation clinic routinely had scleral thickness assessed by using the scleral image mode with enhanced depth imaging (EDI) on the spectral domain AS-OCT (Spectralis software, version 6.3.2.0; Heidelberg Engineering) to optimize the visualization of the sclera. Patients with inactive scleritis were evaluated and included in the study, with the affected areas marked on a scleral drawing diagram completed by a single investigator (PS), as shown in Fig. 1. The same area of the contralateral unaffected eye was imaged to serve as a control and was also marked on the scleral drawing diagram. All AS-OCT images of the affected and the contralateral unaffected sclera were subsequently acquired by a single experienced OCT examiner (SL) following the designated areas marked on the scleral drawing diagram. To accurately measure an area, participants were asked to fixate on a target to ensure that the orientation of the desired area was perpendicular to the incoming light wave from the AS-OCT.

One image per each eye’s anterior sclera were obtained by using AS-OCT volume scans, all of which consisted of at most 25 scans of B-scan with each line raster separated vertically by 277 µm [10–12]. The anterior scleral thickness was taken as the axial distance between the posterior conjunctival boundary and the posterior scleral boundary. Both AS-OCT images were measured at a standardized distance of 2 mm posterior to the limbus by two independent masked graders (JT and MC) using the caliper tool in the Heidelberg OCT software.

We used a standardized data collection sheet which identified the affected and contralateral unaffected sclera on a scleral drawing diagram for each patient. The analyzed images were enlarged up to 800% for precise measurements [11]. We used *en face* infrared reflectance images to locate the area of interest to obtain more accurate anterior scleral thickness measurement. Only high-quality images with accurate depiction of the entire sclera from the 25 axial thickness images were included in the analysis as determined by a uveitis specialist (PS) before being provided to the two masked graders. High-quality scans were defined as those allowing for the identification of the border of posterior conjunctiva and posterior sclera [11]. If any individual scan was considered of poor quality so as to be unevaluable, each grader was allowed to remove that single scan from their analysis. The overlying conjunctiva was excluded from scleral measurements to obtain a more accurate measurement of the sclera/episcleral complex. The average of every scan measured by both graders of the affected sclera’s anterior thickness was compared to the corresponding contralateral unaffected eye. Agreement between graders was calculated using interclass correlation (ICC).

**Fig. 2:**
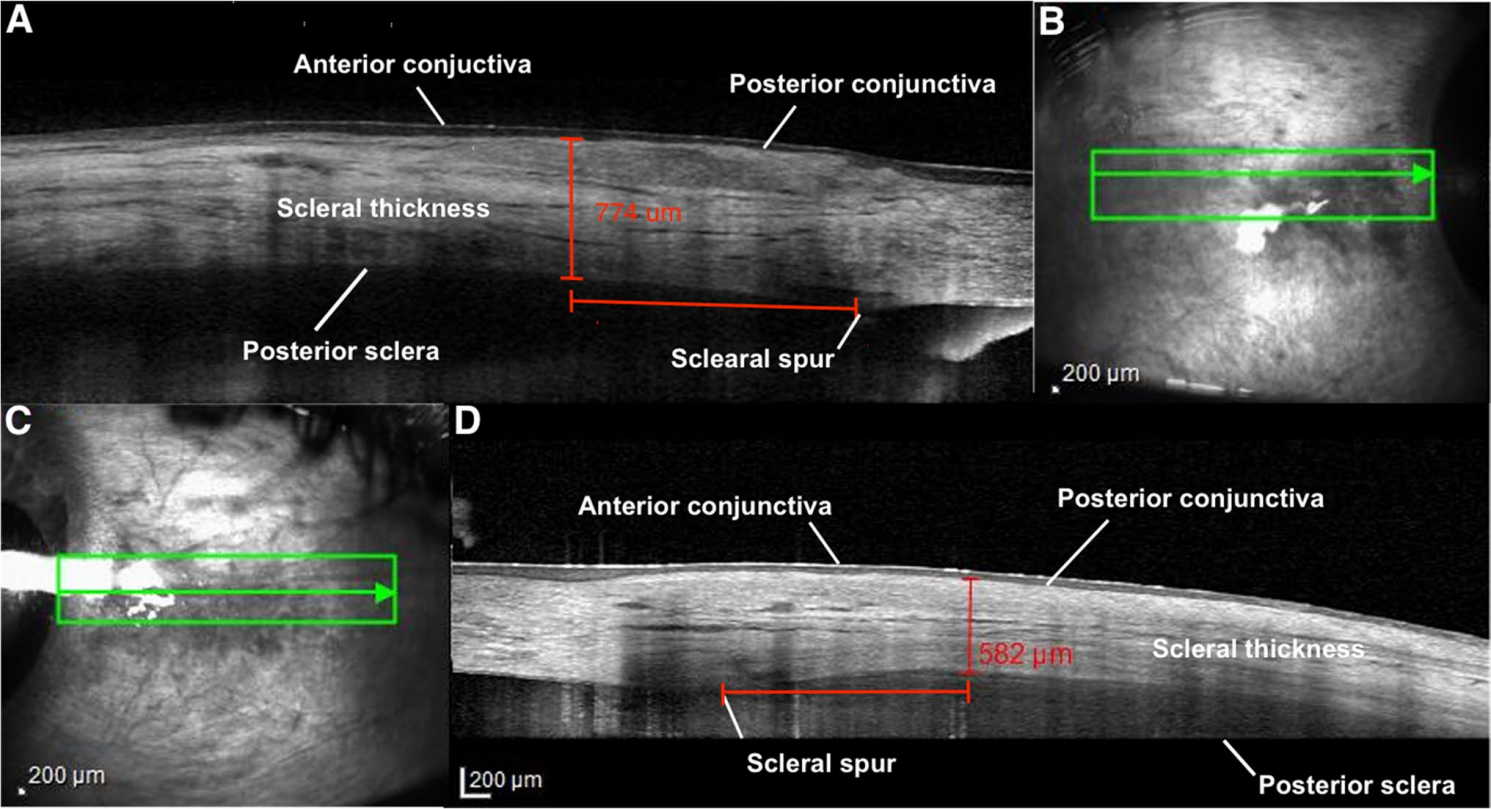
Case 1. Anterior segment optical coherence tomography (AS-OCT) image and image processing of scleral in case 1. **A** The selected infrared scleral image red horizontal line is centered on the 2 mm location from the limbus of contralateral unaffected eye anterior sclera showing a red vertical line measured 774 µm. **B **Infrared image and the scan line (green) of the contralateral unaffected eye anterior sclera. An affected anterior scleral in case 1 was demonstrated in (**C**) Infrared image and the scan line (green) of affected eye anterior sclera. **D **The selected infrared scleral image red horizontal line is centered on the 2 mm location from the limbus of affected sclera showing a red vertical line measured 582 µm

**Fig. 3:**
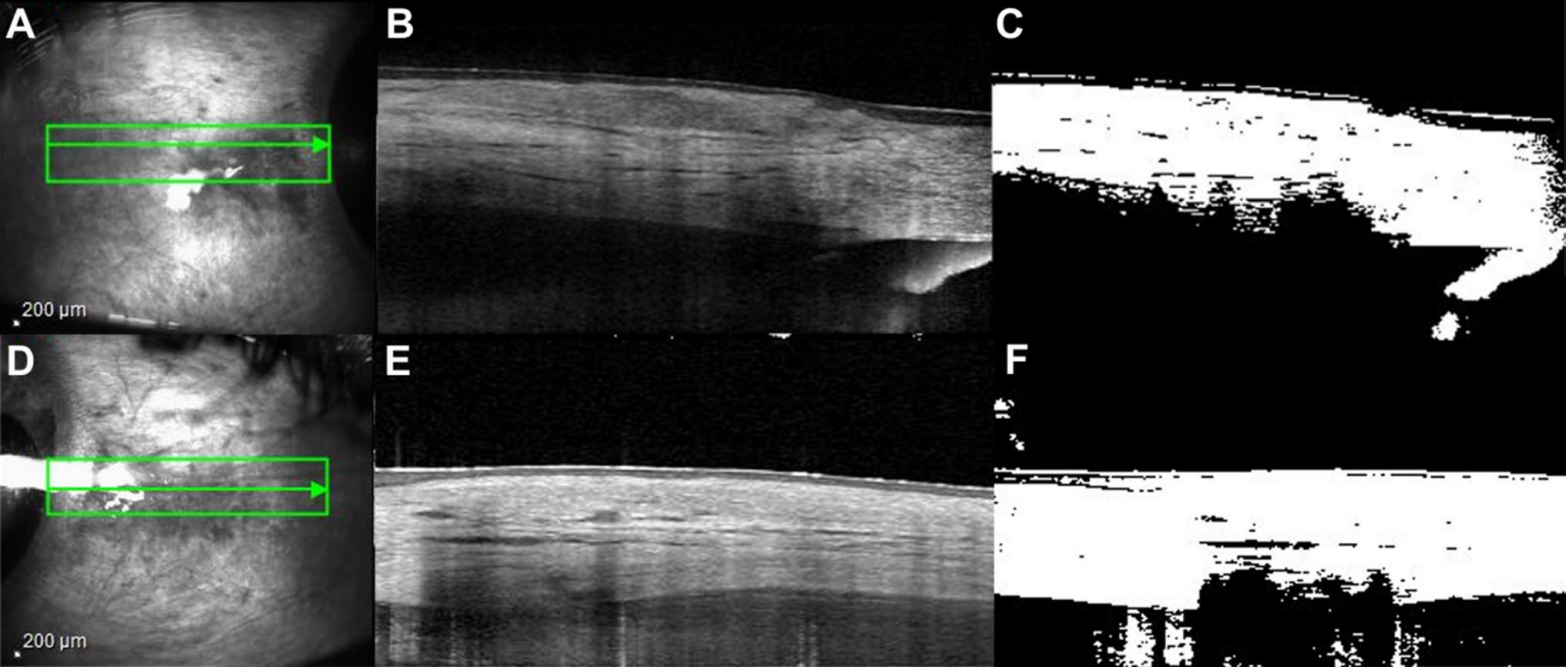
Anterior segment optical coherence tomography (AS-OCT) image of anterior scleral in case 1. **A** Infrared image and the scan line (green) of contralateral unaffected eye anterior sclera (**B**) The selected image of (**A**) then was binarized and computed with a vascular density plugin application using image J (**C**). **D** Infrared image and the scan line(green) of affected sclera. **E** The selected image of (**D**) then was binarized and computed with a vascular density plugin application using image J (**F**)

Scleral hyperreflectivity was assessed using ImageJ software (National Institutes of Health, Bethesda, Maryland, USA). The JPEG-formatted scleral images, exported from the OCT machine, were manually cropped to dimensions of 2 mm vertically and 3 mm horizontally. Each image was centered 2 mm from the limbus (Fig. 2) for both affected and unaffected eyes and the imported into ImageJ as an image stack. The images were then binarized and analyzed with a vascular density plug-in application to determine the image contrast, calculated as the percentage of the white area (indicative of maximum contrast) relative to the overall image area. The percentage of hyperreflective white area in the affected eyes with a violaceous hue was compared to that in the unaffected contralateral eyes (Fig. 3) [13, 14].

## Statistical analysis

Descriptive statistics were used for all nominal and continuous variables and reported with percentage and mean ± standard deviations. Mean scleral thickness of the violaceous areas were compared with the mean scleral thickness of unaffected areas in the corresponding region of the contralateral eyes and analyzed by paired t-test according to variance and distribution. Statistical analyses were conducted with Stata SE18 (StataCorp).

## Results

Nine of 14 patients met the study inclusion criteria. The mean age of patients was 52 ± 12.8 years (range 34 to 67 years) with the majority being female (88.9%). Best-corrected visual acuity (BCVA) ranged from 20/20 to hand motions. None of the patients had significant anisometropia as their refractive errors did not exceed 3 diopters difference between their two eyes as measured by OCT focus settings scanning the macula. All patients demonstrated violaceous areas and were diagnosed with unilateral anterior scleritis (3 in the right eye, and 6 in the left eye) with normal sclera in contralateral eye. Six patients (66.7%) had diffused scleritis and 3 (33.3%) had nodular scleritis. No eyes had active scleritis at time of study inclusion. The most common underlying disease processes associations with scleritis were rheumatoid arthritis (n = 3, 33.3%) and history of treated latent TB (n = 3, 33.3%). Patients with a positive interferon gamma release assay test as part of their scleritis diagnostic evaluation had been previously treated with anti-tuberculous medication. One patient had a diagnosis of granulomatosis with polyangiitis, one with herpes simplex virus, and one with necrobiotic xanthogranuloma. Six patients were being managed with immunosuppressive medication. Detailed demographic data is presented in Table 1.

**Table 1:** Demographic and characteristic of patient with violaceous hue and inactive scleritis

Patient Number	Age/Gender	Affected eye	Type of scleritis	Associated disease	Systemic medications	Topical medications
1	36/F	OD	diffuse	History of treated latent TB	none	PA
2	56/F	OD	nodular	Undifferentiated	MTX 25 mg weekly	none
3	66/F	OS	nodular	GPA/ History of treated latent TB	RTX, Pred 4 mg daily	none
4	58/M	OS	nodular	Undifferentiated	IFX 20 mg/kg q 4 wk, Pred 20 mg daily	Topical NSAID
5	53/F	OS	diffuse	RA	ADA 40 mg q 7 days, Pred 3 mg daily	PA
6	67/F	OS	diffuse	RA	none	none
7	34/F	OS	diffuse	Herpes simplex/ History of treated latent TB	none	none
8	57/F	OS	diffuse	RA	ADA 40 mg q 14 days, MTX 20 mg weekly	FML
9	41/F	OD	diffuse	NXG	IVIG	none

*ADA *adalimumab, *F *female, *FML * fluoromethalone, *GPA *granulomatosis with polyangiitis, *IFX *infliximab, *IVIG *intravenous immunoglobulin, *M *male, *MTX *methotrexate, *NXG *necrobiotic xanthogranuloma, *OD *right eye, *OS *left eye, *PA *prednisolone acetate, *Pred * prednisone, *RA * rheumatoid arthritis, *RTX* rituximab, *TB* tuberculosis, *TCZ *tocilizumab

The mean anterior scleral thickness in affected areas characterized by a violaceous hue was 582.93 µm ± 124.03 µm compared to 648.59 µm ± 103.61 µm in the unaffected contralateral areas (mean difference = −65.65 µm, 95% CI: −143.73 µm to 12.42 µm, *p* = 0.0885). A sensitivity analysis excluding one patient (number 3) with a relatively low number of scans showed mean thicknesses of 569.76 µm ± 125.68 µm and 640.91 µm ± 108.00 µm in the affected and unaffected contralateral area, respectively (mean difference = −71.15 µm, 95% CI: −160.72 µm to 18.43 µm, *p* = 0.1024). The ICC between two graders was 0.929. In 6 of 9 patients, the average thickness of the affected sclera was thinner than the contralateral unaffected sclera. Mean scleral thickness values are shown in Table 2. Exceptions were in one patient with necrobiotic xanthogranuloma-associated scleritis and two patients with undifferentiated scleritis. No AS-OCT images demonstrated characteristics of active scleritis.

**Table 2:** Number of scans (OD,OS) and mean anterior scleral thickness between 2 graders (mean ± SD, um) at 1–3 mm posterior to scleral spur/limbus of the patients (n = 9) in the affected eye and the unaffected eye using anterior segment optical coherence tomography (AS-OCT)

Patient Number	Number of scans (OD,OS)		Scleral thickness (mean ± SD, um)	
	First grader	Second grader	Affected eye	Unaffected eye
1	25,25	25,25	583.26 ± 25.22	789.66 ± 51.13
2	25,25	25,25	716.06 ± 34.50	684.74 ± 36.28
3	3,3	3,9	688.33 ± 23.48	710.00 ± 23.65
4	25,25	25,25	553.68 ± 61.66	652.5 ± 15.39
5	25,25	23,25	621.68 ± 75.45	671.76 ± 43.11
6	25,25	24,25	556.82 ± 38.36	716.71 ± 34.75
7	25,25	14,25	692.08 ± 50.04	647.88 ± 35.02
8	25,25	25,25	306.86 ± 63.99	492.22 ± 16.51
9	25,25	25,25	527.64 ± 75.53	471.78 ± 59.68

*OD *right eye, *OS *left eye, *SD *Standard deviation

The image contrast analysis revealed that the mean percentage of scleral hyperreflectivity was significantly higher in affected areas (65.07 µm ± 6.49 µm) compared to (42.70 µm ± 5.46 µm), with a mean difference = 22.37 µm, 95% CI: 14.72 µm to 30.03 µm, p = 0.0001).

## Discussion

In this descriptive cross-sectional study, we conducted a comparative analysis of scleral thickness and hyperreflectivity in localized regions of the sclera among patients with unilateral anterior scleritis. The regions under assessment exhibited a distinctive violaceous hue characteristic of scleritis without active inflammation. We utilized the contralateral unaffected sclera within the same region as a control for comparison. Our findings indicate that the anterior scleral thickness in violaceous areas of previous scleritis does not significantly differ from the thickness of the unaffected area on the contralateral side. However, these violaceous areas exhibited significantly greater scleral hyperreflectivity compared to their unaffected counterparts. This increased hyperreflectivity may be attributed to collagen remodeling in the affected sclera, resulting in a diminished capacity to absorb violet to blue light wavelengths, thus reflecting these wavelengths and creating the observed violaceous hue.

The scleral stroma is composed primarily of collagen, surrounded by proteoglycans, elastin glycoproteins, and lacks a direct blood supply [15, 16]. Collagen fibers demonstrate a complex interweaving pattern, that gives the sclera its characteristic white color by reflecting most wavelengths of visible light. Histopathological studies have demonstrated degradation of collagen fibers and infiltration of inflammatory cells in scleritis specimens [6, 17]. On an ultrastructural level, the proteoglycans that normally maintain even spacing between collagen fibrils are disrupted during the inflammatory process [6]. Like corneal thinning after keratitis, collagen fibrils show evidence of fraying, which leads to further disorganization of scleral collagen fiber architecture following resolution of active inflammation [6, 18]. This loss of the normal organizational structure likely contributes to both the inflammatory and necrosis properties of the post-inflammatory sclera [19].

Following the resolution of inflammation in non-necrotizing scleritis, the affected sclera may manifest a violaceous hue. Rather from arising primarily from scleral thinning, this characteristic appearance appears to result from the layered organization and spacing of collagen fibers within the sclera [3, 4]. The disruption of dermatan sulfate bonds and fraying of individual collagen fibrils creates irregularities in fiber arrangement that modify how light interacts with the tissue, facilitating selective absorption within the blue to red visible spectrum and thereby producing the observed violaceous hue. In contrast to innate irregularities in collagen fibril diameter and fiber arrangement in non-scleritic sclera (which becomes increasing irregular following scleritis), there can actually be translucency in the sclera when there is more regular arrangement of of collagen fibers as is seen in ostegenesis imperfecta [20, 21]. Our study hypothesized that increased scleral hyperreflectivity on AS-OCT we observed in violaceous areas, compared to unaffected areas, may directly reflect this underlying disorganization of the scleral stroma, compared to unaffected areas, may directly reflect this underlying disorganization of the scleral stroma that has been demonstrated histopathologically [3, 4]. Although most violaceous areas were thinner, the choroid remained substantially thick beneath the imaged surface so that direct visualization of the underlying choroid was not possible.

AS-OCT has been widely employed in the evaluation of corneal and ocular surface conditions [22]. Furthermore, its utilization can identify scleral changes associated with therapeutic responses [23]. Incorporating AS-OCT could serve as a valuable adjunct for measuring scleral thickness in patients with scleritis. AS-OCT offers higher resolution compared to high-frequency ultrasound biomicroscopy, although it may encounter challenges in cases of significant scleral edema due to its limited penetration depth [24]. While existing literature predominantly focuses on AS-OCT's application in healthy eyes or those with active scleritis, there is a notable absence of reports regarding scleral thickness in areas exhibiting inactive violaceous hue in non-necrotizing scleritis [15, 23, 25–27]. Although some reports have documented decreased scleral thickness using AS-OCT post-resolution of scleritis, none have compared it with the corresponding non-scleritic area of the contralateral eye [11, 25].

One strength of our study is the use of a single anterior segment optical coherence tomography (AS-OCT) machine to measure both affected and unaffected scleral areas, which helps to minimize measurement variability. Additionally, the high level of agreement between two masked graders enhances the reliability of our measurements. However, our study has several limitations. The primary limitation is the relatively small sample size. Moreover, our segmentation process required manual delineation of each tissue layer, which could introduce bias, despite the graders being masked to the diagnosis of scleritis. Another limitation is that we were unable to obtain accurate details regarding the number and duration of scleritis attacks, as patients may have underreported or overreported this information due to retrospective recall bias.

## Conclusion

As the difference in scleral thickness of approximately 65 microns did not reach statistical significance in our small sample of nine patients, our findings suggest that violaceous sclera represents collagen remodeling rather than significant thinning. This distinction has important therapeutic implications, indicating that these areas likely maintain structural integrity despite their altered appearance. Clinicians managing patients with resolved scleritis can be reassured that violaceous areas are unlikely to be structurally compromised, reducing concerns about an increased risk of perforation solely based on discoloration.
